# Mother-to-child transmission of HTLV-1:in vitro study of HTLV-1 passage across a tight human epithelial barrier

**DOI:** 10.1186/1742-4690-8-S1-A194

**Published:** 2011-06-06

**Authors:** Sandra Martin-Latil, Nina Gnädig, Adeline Mallet, Marie-Christine Prevost, Marion Desdouits, Olivier Schwartz, Antoine Gessain, Simona Ozden, Pierre-Emmanuel Ceccaldi

**Affiliations:** 1 Unité Epidémiologie et Physiopathologie des Virus Oncogènes, URA CNRS 3015, Institut Pasteur, Paris, France; 2 Plate Forme Microscopie Ultrastructurale, Institut Pasteur, Paris, France; 3 Unité Virus et Immunité, URA CNRS 3015, Institut Pasteur, Paris, France; 4 Université Paris 7, Paris, France

## Background

Besides horizontal transmission, HTLV-1 is transmitted vertically mainly through prolonged breastfeeding. Maternal transmission via breast milk is the dominant mode of HTLV-1 spread in high endemic areas, and is inked to the presence of HTLV-1 infected cells (lymphocytes, epithelial cells..) in the milk. Infection in young childhood by such mean appears to be a major risk factor for development of ATL in adults.

## Materials and methods

We developed an in vitro model of epithelial barrier (Caco-2 human enterocytic cell line) to assess the mode of passage of HTLV-1 through the digestive tract. Integrity of the barrier was checked by ultrastructural approach, measurement of the trans-epithelial resistance (TER), and diffusion of fluorescently labeled molecules.

## Results

When enterocytes were co-cultured with HTLV-1-infected lymphocytes, no structural modifications were detected in intercellular tight junctions. Moreover, the functional integrity of the epithelial barrier was maintained since no TER change was detected in the presence of infected lymphocytes, and passage of small fluorescent markers was unaffected. Although enterocytes were not found to be susceptible to HTLV-1 infection, free infectious HTLV-1 virions were detected in the basal compartment, and such a passage was temperature-dependent, suggesting a transcytotic mechanism of passage. When human dendritic cells were added to the basal compartment, they were found to be productively infected by HTLV-1 that had crossed the epithelial barrier.

## Conclusions

This study provides the first data on the mode of transport of HTLV-1 across a tight epithelial barrier as it may occur during mother-to-child HTLV-1 transmission during breastfeeding.

